# Genome-Wide Association Study of Morphological Defects in Nellore Cattle Using a Binary Trait Framework

**DOI:** 10.3390/genes16101204

**Published:** 2025-10-14

**Authors:** Milena A. F. Campos, Hinayah Rojas de Oliveira, Henrique A. Mulim, Eduarda da Silva Oliveira, Pablo Augusto de Souza Fonseca, Gregorio M. F. de Camargo, Raphael Bermal Costa

**Affiliations:** 1 School of Veterinary Medicine and Animal Science, Federal University of Bahia, Salvador 40170-110, BA, Brazil; mapareci@purdue.edu (M.A.F.C.); gregorio.camargo@ufba.br (G.M.F.d.C.); raphael.bermal@ufba.br (R.B.C.); 2 Department of Animal Sciences, Purdue University, West Lafayette, IN 47907, USA; hmulim@purdue.edu (H.A.M.); eduarda.s.oliveira@unesp.br (E.d.S.O.); 3 School of Agriculture and Veterinary Science, São Paulo State University, Jaboticabal 14884-900, SP, Brazil; 4 Mountain Livestock Institute (CSIC-ULE), 24346 Leon, Spain; p.fonseca@csic.es

**Keywords:** candidate genes, fastGWA-GLMM, Nellore cattle, QTL, structural soundness, threshold trait, zebu

## Abstract

Background/Objectives: Morphological defects such as limb malformations, cranial asymmetries, loin deviations, jaw misalignments, and navel irregularities are associated with early culling and reduced productivity in beef cattle. In *Bos taurus indicus* such as Nellore, the genetic basis of these traits remains poorly characterized. This study aimed to investigate the genetic architecture of six morphological defects in Nellore cattle, namely feet and legs malformation, chamfer asymmetry, fallen hump, loin deviation, jaw misalignment, and navel irregularities, via a genome-wide association study (GWAS) approach tailored for binary traits. Methods: Depending on the trait, the number of genotyped animals analyzed ranged from 3369 to 23,206, using 385,079 SNPs (after quality control). Analyses were conducted using a linear mixed model framework adapted for binary outcomes. Results: Significant associations were identified for four traits: feet and legs, chamfer, hump, and loin. No significant markers were detected for jaw or navel defects, likely due to lower sample sizes and trait incidence. Gene annotation revealed 49 candidate genes related to feet and legs, 4 for chamfer, 4 for hump, and 6 for loin. Conclusions: Candidate genes were enriched for biological functions, including bone remodeling, muscle development, lipid metabolism, and epithelial organization. Overlaps with QTL related to conformation, feed intake, reproductive traits, and carcass quality were also observed. These findings provide novel insights into the genetic control of morphological defects in Nellore cattle and may inform breeding strategies aimed at improving structural soundness.

## 1. Introduction

Morphological defects in cattle, including limb malformations, cranial asymmetries, loin deviations, jaw misalignments, and navel irregularities, can compromise animal welfare, productivity, and longevity [1]. These defects often lead to early culling and economic losses for producers and are undesirable in breeding programs where structural soundness is a primary selection criterion [2,3]. Although their population incidence is generally low (<10%; [4]), the impact of these defects is disproportionate, as they may impair locomotion, reproductive performance, and adaptability to extensive production systems, thereby reducing overall herd efficiency [3,5].

Nellore cattle, the predominant beef breed in Brazil, form the foundation of the national herd and are widely used in extensive, pasture-based systems due to their adaptability and efficiency in tropical environments [6,7,8]. Because these cattle must withstand high temperatures, navigate varied terrains, and travel long distances to access resources, maintaining structural soundness is essential for productivity, longevity, and animal welfare. Consequently, morphological defects that impair mobility or conformation can have direct economic and welfare impacts, underscoring the importance of understanding their genetic basis in breeding programs. Nonetheless, the genetic basis of specific morphological defects remains poorly understood in Nellore cattle. While leg and foot issues have received some attention in the literature because of their obvious impact on locomotion and productivity, other structural conditions, such as cranial asymmetry (chamfer), fallen hump, loin defects, jaw misalignment, and navel abnormalities, are rarely studied in terms of inheritance patterns and genetic architecture. Moreover, these traits are often under-recorded in routine evaluations, partly because of their subjective assessment and the difficulty of standardizing scoring across farms [3,9].

The binary nature of these traits (i.e., presence or absence), combined with their low frequency and environmental influences, presents unique statistical challenges, as traditional linear models may not be appropriate for analyzing such data, requiring specialized analytical approaches that can properly account for the underlying liability distribution. Despite this, previous research suggests that morphological traits may have moderate heritability [2], indicating that a genetic component controls these traits. In addition, other related traits, such as muscularity scores, have high heritability estimates in Nellore cattle (~0.38; [10]). Recent studies have also shown that taurine gene introgression has been linked to growth and structural variation in zebu populations, suggesting that genetic diversity and breed composition can influence morphological development [11].

The development of high-density single-nucleotide polymorphism (SNP) arrays and imputation tools has increased our ability to explore the genetic architecture of complex traits in livestock, providing unprecedented resolution for identifying genomic regions associated with phenotypic variation [12]. Methods tailored for binary traits allow for the analysis of large datasets while accounting for population structure and relatedness, enabling the detection of genomic regions associated with rare or under-recorded conditions [13,14,15]. However, very few GWASs have investigated morphological defects in *Bos indicus* cattle, and none have specifically applied such binary-trait approaches [3,16]. Expanding the knowledge on the genetic basis of morphological defects is essential to support more informed breeding decisions, reduce undesired phenotypes, and promote structural integrity in selection candidates. Genomic information could also reveal shared biological pathways or pleiotropic effects relevant to broader aspects of soundness and productivity. Therefore, the objectives of this study were to: (1) investigate the genetic architecture of six morphological defects in Nellore cattle (i.e., feet and legs malformation, chamfer asymmetry, fallen hump, loin deviation, jaw misalignment, and navel irregularities) using the fastGWA-GLMM approach; (2) annotate candidate genes and QTLs associated with the morphological defects; and (3) explore the biological pathways involved in the significant genomic regions associated with these defects.

## 2. Materials and Methods

The data analyzed were provided by the Gensys^®^ company (Porto Alegre, Rio Grande do Sul, Brazil) through the DeltaGen^®^ breeding program, which performs genetic evaluations of Nellore cattle raised in Brazil. As these data originated from existing routine evaluations, approval from an animal care and use committee was not required.

### 2.1. Phenotypic Data

Phenotypic records were collected between 1999 and 2023 during three evaluation stages: weaning (approximately 7 months of age), yearling (16 months of age), and final evaluation (18 months of age). In these evaluations, trained professionals measured growth and reproductive traits, scored visual assessment of conformation, precocity, muscularity, and navel condition (CPMU), and identified the presence of morphological defects. The original dataset included records for 799,672 animals, including those with and without the defect of interest. Morphological defects were visually assessed via a binary approach (i.e., 1 for presence and 0 for absence of the defect). Contemporary groups (CG) were defined based on birth year and season, sex, farm, and management group at weaning and yearling, as well as date of measurement at weaning and yearling. Groups with fewer than 10 animals or without variation in the trait were discarded. Connectedness among CG was verified using AMC software Version 4.1 [17], and disconnected groups were excluded.

### 2.2. Genomic Information

Genotypes were available for 68,859 animals. These animals were originally genotyped using Neogen^®^’s 50K SNP panel [18] and subsequently imputed to the 777K density using the Illumina Bovine HD array (Illumina, San Diego, CA, USA) [19]. Imputation was performed as part of Gensys^®^’s official evaluation system using the FImpute V3 software [20], relying on a reference population of 6105 core Nellore samples with imputation accuracies above 0.97 [21]. For the GWAS, only animals with both phenotype and genotype information were included. Quality control (QC) of genotypes was performed for each trait using PLINK [22]. SNPs were retained if they met the following thresholds: call rate > 0.98, minor allele frequency (MAF) > 0.05, and Hardy–Weinberg equilibrium *p* < 10^−5^. Animals with an individual call rate < 0.99 were also removed. Table 1 summarizes the final number of animals included in the GWAS for each trait and the incidence of defects in the analyzed sample (i.e., population of animals with both genotypes and phenotypes).

The number of animals used in the GWAS varied by trait, as only animals with both genotypes and phenotypes were used. The incidence was calculated as the proportion of affected animals among those evaluated.

### 2.3. Genome-Wide Association Studies

The genome-wide association analyses were carried out using the ultrafast generalized linear mixed model for binary traits (fastGWA-GLMM, [14]) implemented in the GCTA software v1.94.1 [15]. This framework extends the fastGWA algorithm by incorporating a generalized linear mixed model (GLMM), and applies sparse relationship matrix (GRM) to efficiently estimate parameters and perform association tests [14]. The fitted model is defined as follows:

$$logit\left(\mu \right)=x_{s}\beta_{s}+X_{c}\beta_{c}+g,$$

where

y is a n×1 vector of binary phenotypes;
μ is a vector of $\mu_{i}=P\left(y_{i}=1|x_{si},X_{ci},g_{i}\right)$ with $\mu_{i}$ representing the probability of an individual i being a case given their genotype $x_{si}$, fixed effects (contemporary groups) $X_{ci}$, and the animals were used as a random genetic effect $g_{i}$.
$x_{s}$ is a vector of genotypes of a variant of interest with its effect $\beta_{s}$,
$X_{c}$ is the incidence of contemporary groups used as a fixed effect with their corresponding coefficients $\beta_{c}$.
g is a vector of effects that captures genetic and common environment effects shared among related individuals, $g∼N\left(0,\pi \sigma_{g}^{2}\right)$ with π being the sparse GRM (i.e., GRM with all the small off-diagonal elements set to zero), and $\sigma_{g}^{2}$ being the corresponding variance component.

The fastGWA-GLMM method consists of two main steps. First, parameter estimation is performed via a computationally efficient grid search-based algorithm [14]. During this process, the genetic variance component $\sigma_{g}^{2}$ and residual variance component $\sigma_{e}^{2}$ are estimated by maximizing the likelihood under the logistic regression model. The grid search explores a range of possible values for these variance components, and the combination that maximizes the likelihood is selected. Genetic variance is estimated by modeling covariance through the (GRM), while the residual variance is treated as part of the error term [14]. Second, association testing is performed via a score test for each variant, i.e.,

$$T_{score}=x_{s}^{T}\left(y-\mu \right)\;with\;var\left(T_{score}\right)=x_{s}^{T}Px_{s},$$

$$\frac{T_{score}^{2}}{var\left(T_{score}\right)}∼\chi_{d.f.=1}^{2},$$

where $P=V^{-1}-V^{-1}X_{c}{\left(X_{c}^{T}V^{-1}X_{c}\right)}^{-1}X_{c}^{T}V^{-1}\;with\;V=W^{-1}+\pi \sigma_{g}^{2}$ and **W** is a diagonal matrix, i.e., $W_{ii}\;=\;\mu_{i}\left(1-\mu_{i}\right)$. The P is an n×n projection matrix, which is dense despite π being sparse.

Finally, multiple-testing correction was applied using the Bonferroni method (α = 0.05), with the number of independent chromosome segments estimated according to the length of each chromosome and the effective population size (Ne = 196) [23,24]. This approach adjusts the SNP significance thresholds while accounting for linkage disequilibrium and population structure [25].

### 2.4. Gene Annotation, QTL Identification, and Functional Enrichment Analyses

The significant markers associated with the phenotypic defects were retained for gene annotation, QTL annotation, and functional enrichment analysis. The annotation of genes and QTLs was performed using the GALLO package [26] available in R [27]. For that, a window of 100 Kb up and downstream from the significant SNP marker was used. After annotation, the positional candidate genes were analyzed through functional enrichment of Gene Ontology (GO) terms, including biological processes (BP), metabolic functions (MF), and cellular components (CC), as well as the Kyoto Encyclopedia of Genes and Genomes (KEGG) pathways using the gprofiler2 package [28].

## 3. Results

### 3.1. Genome-Wide Association Studies

**Figure 1:**
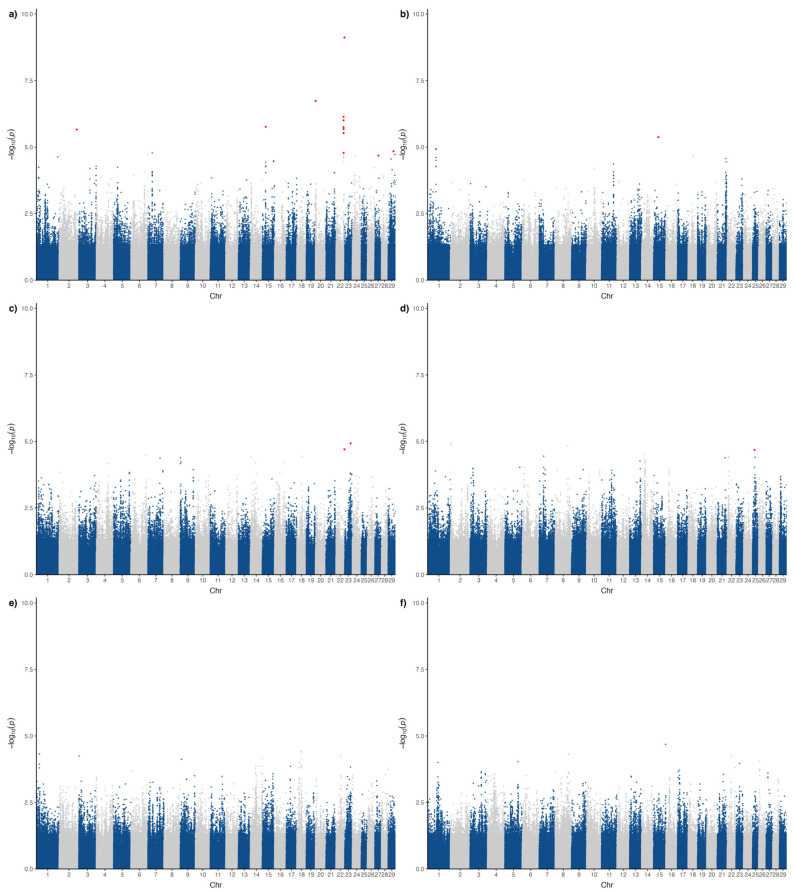
Manhattan plots for (**a**) feet and legs malformation, (**b**) chamfer asymmetry, (**c**) fallen hump, (**d**) loin deviation, (**e**) jaw misalignments, and (**f**) navel irregularities. Significant markers are highlighted with red dots.

Significant associations were detected for four traits: feet and legs malformation, chamfer asymmetry, fallen hump, and loin deviation (Figure 1). No genome-wide significant markers were found for jaw misalignment or navel irregularities, likely due to lower incidence and sample sizes. The QQ-plots for all analyzed traits are shown in the Supplementary Material Figure S1. The genomic inflation factors (λ) ranged from 1.06 (jaw) to 1.22 (feet and legs), suggesting control for population structure while maintaining sensitivity to detect true associations.

For feet and legs malformation trait, 13 significant markers were identified across six chromosomes (Figure 1a), with a strong cluster on BTA22. Chamfer asymmetry was associated with a single SNP on BTA15 (Figure 1b), while fallen hump was associated with markers on BTA22 and BTA23 (Figure 1c), including one overlapping signal with feet and legs. Loin deviation was associated with a single SNP on BTA25 (Figure 1d).

### 3.2. Gene Annotation, QTL Identification, and Functional Enrichment Analyses

**Figure 2:**
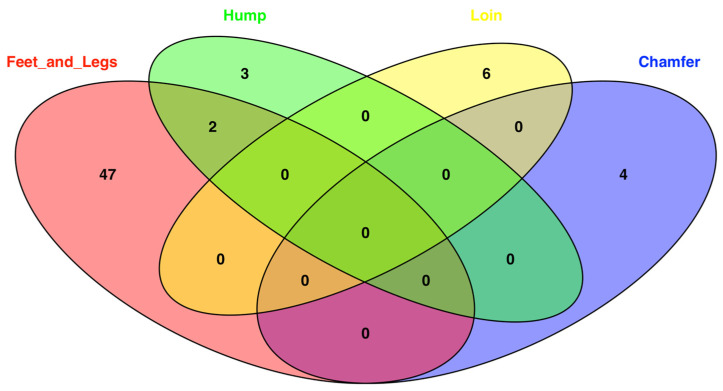
Venn diagram illustrating the overlap of genes identified for each trait (feet and legs malformation, chamfer, hump, and loin) in Nellore cattle. Each circle represents a set of genes associated with a specific trait, with the intersections indicating genes shared across traits.

Gene annotation within 100 kb windows around significant markers revealed distinct sets of candidate genes for each trait (Table 2). providing insights into the potential biological mechanisms underlying these morphological defects. The number of positional candidate genes overlapping among traits is shown in Figure 2.

Table 2 shows the most likely candidate genes identified for each trait. The complete lists of candidate genes are provided in Supplementary Material Tables S1–S4.

Only feet and legs malformation and hump traits share two common genes (*IQSEC1* and *ACAD9*). This overlap suggests a potential genetic relationship between these two traits, which may warrant further investigation to understand the underlying genetic mechanisms controlling feet and legs malformation and hump traits. Table 3 shows the main associated GO terms and functional annotations for each trait; the full GO table is presented in Supplementary Materials Tables S5–S8.

Feet and legs malformation showed 49 candidate genes, including several chemokine receptor genes (*CCR1*, *CCR2*, *CCR3*, *CCR5*, *CCRL2*) and *SLIT3*, a gene implicated in skeletal development. Chamfer asymmetry highlighted *GRAMD1B* and *CLMP*, which are involved in cholesterol metabolism and cell adhesion. Hump-associated markers mapped to genes such as *IQSEC1* and *ACAD9*, linked to signal transduction and fatty acid metabolism. Loin deviation revealed *MYH11*, *NDE1*, *MARF1*, and microRNAs (bta-mir-484, bta-mir-1246), suggesting roles in cytoskeletal organization and muscle biology. Functional enrichment analyses revealed processes related to immune signaling, chemotaxis, and cell adhesion for feet and legs malformation, consistent with connective tissue and locomotor functions. Chamfer asymmetry was associated with cholesterol-related pathways, while hump was linked to signal transduction and energy metabolism. Loin deviation enrichment pointed to cytoskeletal organization and regulation of cell motility. Interestingly, QTL annotation (Table 4) showed overlaps between associated regions and previously reported loci for traits such as body weight, calving ease, feed efficiency, carcass quality, and health traits, highlighting potential links between structural soundness and broader aspects of productivity and adaptation.

## 4. Discussion

To the best of our knowledge, this study represents the first comprehensive genome-wide association analyses targeting visually assessed morphological defects in *B. indicus* cattle using a model specifically designed for binary traits. By applying fastGWA-GLMM, we identified significant genomic regions for feet and legs malformation, chamfer asymmetry, fallen hump, and loin deviation, providing new insights into the genetic control of structural soundness in Nellore cattle.

### 4.1. Methodological Aspects and Statistical Considerations

The number of animals included in the analyses varied substantially across traits (Table 1), which strongly influenced the ability to detect associations. Traits such as feet and legs and chamfer, with >22,000 animals, provided greater statistical power, while jaw and navel, with fewer records, were less likely to yield significant loci. Additionally, the low incidence of these defects (3.44–8.69%) also poses challenges for binary-trait GWAS. The genomic inflation factors observed (λ = 1.08–1.22; Supplementary Figure S1) are slightly above the ideal but remain within the range reported for highly polygenic traits analyzed in large datasets and high-density marker panels [29]. As noted by Gurinovich et al. [27], fastGWA-GLMM can appear overly conservative when applied to family-based data, and λ values between 1.1 and 1.2 may reflect true polygenic signals rather than population stratification or cryptic relatedness. In this context, the QQ-plots without lambda correction showed better visual alignment with the expected distributions compared to the corrected versions, suggesting that the observed inflation may reflect genuine polygenic architecture rather than methodological artifacts. This phenomenon has been previously reported in GWAS using similar analytical frameworks, where overcorrection can lead to an excess of false negatives [13]. The use of sparse genomic relationship matrices (GRM) in the fastGWA-GLMM framework, rather than the traditional dense GRM, may contribute to these patterns while maintaining computational efficiency for large datasets. Therefore, while fastGWA is computationally efficient and robust in large-scale analyses, it may have reduced sensitivity in detecting associations for rare binary outcomes owing to model approximation and case–control imbalance. More studies comparing the suitability of this approach for family-based related data are needed. Nevertheless, even traits with low frequency, such as morphological defects, can have substantial economic and welfare consequences, justifying genetic investigations.

### 4.2. Genetic Architecture and Biological Mechanisms of Morphological Defects

The strongest association signals were observed for feet and legs malformation, particularly on BTA22, where a cluster of CC chemokine receptor genes (*CCR1*–*CCR5*, *CCRL2*) was detected. These genes are key regulators of immune cell recruitment and bone remodeling, both fundamental for skeletal development. Experimental studies have shown that *CCR3* influences cortical bone thickness by modulating osteoclast and osteoblast activity, although with sex-specific effects [30], suggesting that genetic variation in this region may affect limb structure and joint stability. The nearby *SLIT3* gene encodes a secreted signaling molecule required for osteoblast differentiation and bone matrix organization [31]. Together, these findings support a mechanism where altered immune signaling and bone remodeling pathways contribute to variation in limb conformation and structural soundness. Additional candidate genes, such as *LCK*, *CD5*, and *CD6*, participate in immune regulation [32], reinforcing the role of immune–skeletal interactions.

For chamfer asymmetry, significant associations were found near *GRAMD1B* and *CLMP*, both implicated in craniofacial development. The *GRAMD1B* encodes a cholesterol-sensing protein that regulates lipid trafficking, membrane organization, and autophagy [33]. Disruption of these processes impairs cell differentiation and craniofacial morphogenesis [34,35]. In cattle, *GRAMD1B* has also been linked to fertility and carcass traits [36], suggesting broader biological relevance. *CLMP*, which contributes to tight-junction formation and epithelial adhesion [37], may further affect epithelial integrity during facial development. Together, these results suggest that defective epithelial organization and cellular signaling during craniofacial morphogenesis may underlie chamfer asymmetry in Nellore cattle.

In the case of the fallen hump, significant loci included *ACAD9* and *IQSEC1*, both related to energy metabolism and body composition. *ACAD9* encodes a mitochondrial enzyme essential for β-oxidation and Complex I assembly [38]. Deficiencies in *ACAD9* cause muscle weakness and exercise intolerance in mouse and human models [39,40], while in yaks, related dehydrogenases are differentially expressed under cold-induced metabolic stress [41]. *IQSEC1* has been linked to fat mass distribution in humans using a large-scale GWAS meta-analyses [42]. Considering that the hump is a muscle and fat rich structure, variation in these genes could influence its formation or maintenance, supporting a biological connection between muscular energetics, adiposity, and hump morphology. Functional enrichment also indicated lipid metabolism and intracellular signaling pathways consistent with this interpretation.

For loin deviation, the most plausible candidate was *MYH11*, which encodes a smooth muscle myosin heavy chain essential for cytoskeletal integrity and contractile function [43]. Alterations in *MYH11* can disrupt fiber organization and tissue alignment, potentially contributing to deviations in loin structure. Nearby genes such as *NDE1*, involved in microtubule organization [44], and bta-mir-484, which regulates mitochondrial function and skeletal muscle differentiation [45], may add regulatory layers influencing tissue architecture.

Although limited overlap across traits was detected, *ACAD9* and *IQSEC1* appeared in more than one analysis, suggesting possible pleiotropic effects connecting structural development and metabolic regulation. Overall, the biological functions of these genes indicate that morphological defects in Nellore cattle are influenced by pathways related to bone remodeling [30,31], epithelial and craniofacial development [33,34,35,37], muscle energetics [38,39,40,43], and fat deposition [42], supporting a polygenic and multifactorial architecture underlying structural soundness.

### 4.3. Limitations and Future Research Directions

Several limitations should be acknowledged in this study. For instance, while the binary nature of phenotypic assessment is practical for field conditions, it may not capture the full spectrum of morphological variation. The subjective nature of visual assessment, despite training protocols, introduces potential variability in trait definition and classification, which may compromise the identification of the genomic backgrounds controlling the traits. Additionally, the relatively low incidence of some defects (particularly jaw and navel) limits the statistical power to detect associations for these traits. Future research should consider implementing more objective measurement approaches where feasible, such as photogrammetric analysis or standardized scoring systems with multiple evaluators. The validation of identified associations in independent populations would strengthen the evidence for these genomic regions. Furthermore, functional studies investigating the specific roles of candidate genes in relevant developmental processes could provide deeper insights into the biological mechanisms underlying these defects.

## 5. Conclusions

This study provides the first genome-wide investigation of visually assessed morphological defects in *B. indicus* cattle. We identified key genomic regions associated with feet and legs, cranial asymmetry, hump, and loin, highlighting candidate genes such as *SLIT3* and chemokine receptors on BTA22, *GRAMD1B* for craniofacial asymmetry, *ACAD9* and *IQSEC1* for hump, and *MYH11* for loin structure. These findings reveal that immune signaling, lipid metabolism, and cytoskeletal regulation contribute to the manifestation of structural defects. Although individually rare, such defects carry important welfare and economic implications. The genomic regions identified here provide a foundation for incorporating structural soundness into breeding programs, particularly through genomic selection and marker-assisted management. Future validation in independent populations and functional characterization of key candidates will be essential to translate these discoveries into practical tools for improving herd robustness, productivity, and sustainability.

## Figures and Tables

**Table 1:** Summary of the sample size and incidence of each morphological defect evaluated in this study.

Trait	Number of Animals	Number of Animals with Defects	Incidence (%)
Feet and legs	22,493	1954	8.69
Chamfer	23,206	1053	4.54
Hump	9779	439	4.49
Loin	15,225	566	3.72
Jaw	9077	312	3.44
Navel	3369	155	4.60

**Table 2:** Functional candidate genes identified for each trait.

Gene Name	Chromosome	Genomic Region (bp)		Ensembl Gene ID
		Start	End	
Feet and legs				
*LCK*	2	121,262,594	121,283,528	ENSBTAG00000012695
*USP28*	15	24,336,893	24,400,181	ENSBTAG00000002323
*SLIT3*	20	447,017	1,163,732	ENSBTAG00000017746
*CCR1*	22	53,199,437	53,237,483	ENSBTAG00000019428
*CCRL2*	22	52,998,319	53,000,315	ENSBTAG00000006155
*CCR5*	22	53,024,929	53,032,609	ENSBTAG00000067584
*CCR2*	22	53,041,056	53,057,320	ENSBTAG00000056962
*CCR3*	22	53,134,643	53,166,540	ENSBTAG00000001338
*CD6*	29	37,311,550	37,357,284	ENSBTAG00000018367
*CD5*	29	37,422,886	37,444,466	ENSBTAG00000013730
Chamfer				
*GRAMD1B*	15	33,964,574	34,225,319	ENSBTAG00000001410
*CLMP*	15	33,689,099	33,794,265	ENSBTAG00000020046
Hump				
*IQSEC1*	22	58,592,571	58,878,769	ENSBTAG00000003237
*ACAD9*	22	58,892,389	58,940,024	ENSBTAG00000003242
*ATXN1*	23	40,688,296	40,830,587	ENSBTAG00000019675
*GMPR*	23	40,838,557	40,899,426	ENSBTAG00000015743
Loin				
*MARF1*	25	14,044,148	14,083,629	ENSBTAG00000020387
*NDE1*	25	14,084,964	14,144,211	ENSBTAG00000015986
*MYH11*	25	14,124,964	14,277,425	ENSBTAG00000015988
*BMERB1*	25	13,881,271	14,026,628	ENSBTAG00000011692

**Table 3:** The main significant Gene Ontology (GO) terms identified from candidate genes associated with defects in Nellore cattle.

GO Identification	Category	*p*-Value	Term	Ensembl Gene ID
Feet and legs				
GO:0070098	GO:BP	3.89 × 10^−6^	chemokine-mediated signaling pathway	ENSBTAG00000017746, ENSBTAG00000019428, ENSBTAG00000031355, ENSBTAG00000056962, ENSBTAG00000001338
GO:0006935	GO:BP	1.74 × 10^−3^	chemotaxis	ENSBTAG00000017746, ENSBTAG00000019428, ENSBTAG00000031355, ENSBTAG00000056962, ENSBTAG00000001338
GO:0006955	GO:BP	1.93 × 10^−3^	immune response	ENSBTAG00000001292, ENSBTAG00000019428, ENSBTAG00000031355, ENSBTAG00000056962, ENSBTAG00000001338, ENSBTAG00000018367, ENSBTAG00000019015, ENSBTAG00000048470
KEGG:04514	KEGG	8.39 × 10^−3^	Cell adhesion molecules	ENSBTAG00000019486, ENSBTAG00000039149, ENSBTAG00000018367
Chamfer				
GO:0015485	GO:MF	0.009	cholesterol binding	ENSBTAG00000001410
GO:0071397	GO:BP	0.024	cellular response to cholesterol	ENSBTAG00000001410
Hump				
GO:0032011	GO:BP	0.049	ARF protein signal transduction	ENSBTAG00000003237
GO:0051791	GO:BP	0.049	medium-chain fatty acid metabolic process	ENSBTAG00000003242
GO:0003920	GO:MF	0.014	GMP reductase activity	ENSBTAG00000015743
Loin				
GO:0097435	GO:BP	0.018	supramolecular fiber organization	ENSBTAG00000015986, ENSBTAG00000015988, ENSBTAG00000011692
GO:0031109	GO:BP	0.018	microtubule polymerization or depolymerization	ENSBTAG00000015986, ENSBTAG00000011692
GO:0021822	GO:BP	0.018	negative regulation of cell motility involved in cerebral cortex radial glia guided migration	ENSBTAG00000011692

BP: Biological Process; MF: Molecular Function; KEGG: Kyoto Encyclopedia of Genes and Genomes pathway.

**Table 4:** QTLs overlapping with candidate genomic regions associated with morphological defects in Nellore cattle.

Chromosome	SNP ID	Position (bp)	QTL Type	Name
Feet and legs				
15			Meat and Carcass	Shear force; Marbling score
	rs41746697	24,243,265	Production	Body weight
			Reproduction	Pregnancy rate; Conception rate
20	rs133818511	1,067,779	Production	Body depth
			Reproduction	Calving ease; Pregnancy rate
			Exterior	Foot angle; Feet and leg conformation; Udder attachment; Stature; Strength
			Production	Length of productive life; Methane production
			Health	Somatic cell score
22	rs137317872	52,912,130	Meat and Carcass	Connective tissue amount
			Health	Bovine respiratory disease susceptibility; Clinical mastitis
22	rs136044991	58,804,102	Meat and Carcass	Muscle taurine content
			Production	Body depth; Body weight
27	rs135251990	24,315,347	Meat and Carcass	Marbling score
29	rs42183554	37,423,894	Meat and Carcass	Tenderness score
Chamfer				
15	rs136075448	33,880,728	Production	Dry matter intake
			Meat and Carcass	Marbling score
Hump				
22	rs136044991	58,804,102	Meat and Carcass	Muscle taurine content
			Production	Body depth; Body weight
			Health	Bovine respiratory disease susceptibility
23	rs134164538	40,775,703	Meat and Carcass	Marbling score; Shear force
			Reproduction	Inseminations per conception; Interval to first estrus after calving
Loin				
25	rs137228331	14,067,719	Production	Dry matter intake; Residual feed intake

## Data Availability

The data/models analyzed during the current study are not publicly available due to commercial confidentiality agreements with the data provider, Gensys^®^. Data may be available from Diercles Francisco Cardoso (gensys.diercles@gmail.com) on reasonable request and with permission of Gensys^®^.

## Supplementary Materials

The following supporting information can be downloaded at: https://www.mdpi.com/article/10.3390/genes16101204/s1, Figure S1: QQ-plots from the morphological defects (a) feet and legs malformation; (b) chamfer; (c) hump; (d) loin; (e) jaw, and (f) navel; Table S1: Positional candidate genes identified for feet and legs malformation; Table S2: Positional candidate genes identified for chamfer defect trait; Table S3: Positional candidate genes identified for hump defect trait; Table S4: Positional candidate genes identified for the loin defect trait; Table S5: Significant Gene Ontology (GO) terms (FDR-adjusted *p*-values < 0.05) identified from candidate genes associated with feet and legs malformation in Nellore cattle; Table S6: Significant Gene Ontology (GO) terms (FDR-adjusted *p*-values < 0.05) identified from candidate genes associated with chamfer defect in Nellore cattle; Table S7: Significant Gene Ontology (GO) terms (FDR-adjusted *p*-values < 0.05) identified from candidate genes associated with hump defect in Nellore cattle; Table S8: Significant Gene Ontology (GO) terms (FDR-adjusted *p*-values < 0.05) identified from candidate genes associated with loin defect in Nellore cattle.

## Abbreviations

The following abbreviations are used in this manuscript:

AMC	A computer program to assess the degree of connectedness among contemporary groups
ASIP	Agouti signaling protein
BTA	*Bos taurus* autosome
bp	Base pairs
CC	Cellular Component (Gene Ontology domain)
CG	Contemporary group
CCR	C-C motif chemokine receptor
CLMP	CXADR-like membrane protein
FDR	False discovery rate
GALLO	Genomic Annotation in Livestock for positional candidate Loci (R package)
GCTA	Genome-wide Complex Trait Analysis
GLMM	Generalized linear mixed model
GO	Gene Ontology
GO:BP	Gene Ontology Biological Process
GO:CC	Gene Ontology Cellular Component
GO:MF	Gene Ontology Molecular Function
GRM	Genomic relationship matrix
GWAS	Genome-wide association study
HD	High density
Kb	Kilobase pairs
KEGG	Kyoto Encyclopedia of Genes and Genomes
MAF	Minor allele frequency
Ne	Effective population size
QC	Quality control
QTL	Quantitative trait locus
SNP	Single-nucleotide polymorphism
