# Supplementary material for: Genome-Wide Association Study of Morphological Defects in Nellore Cattle Using a Binary Trait Framework
Source: Genes (Basel). 2025 Oct 14;16(10):1204. doi: 10.3390/genes16101204 (PMC12562650; doi:10.3390/genes16101204)
Supplement: Supplementary file 1 [file genes-16-01204-s001.zip › genes-3874315-supplementary.pdf]

Supplementary Materials

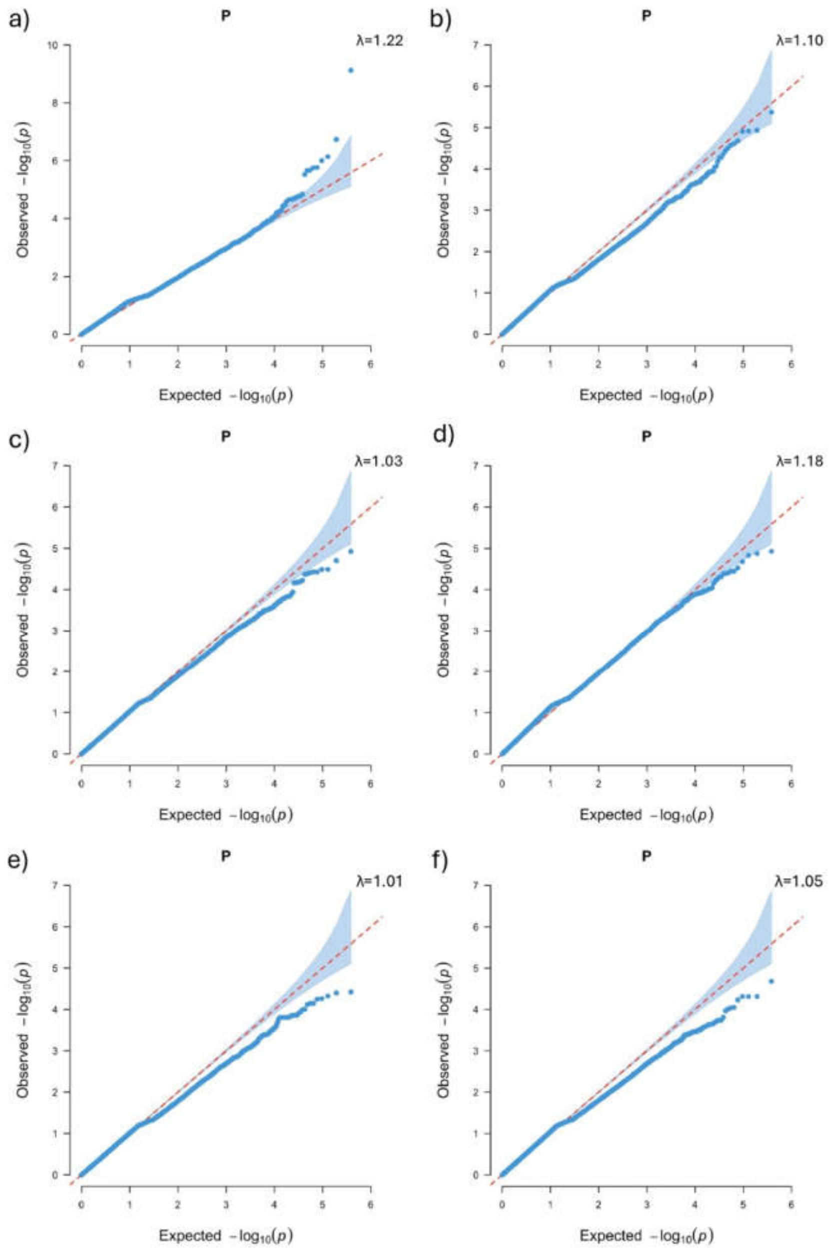

Figure S1. QQ-plots from the morphological defects a) feet and legs malformation; b) chamfer; c) hump; d) loin; e) jaw, and f) navel.

Table S1. Positional candidate genes identified for feet and legs malformation

Gene	Chromosome	Genomic Region (Start-End)		gene_id	gene_biotype
LCK	2	121262594	121283528	ENSBTAG00000012695	protein_coding
FAM167B	2	121285365	121288233	ENSBTAG00000002027	protein_coding
MTMR9L	2	121291097	121298198	ENSBTAG00000014390	protein_coding
EIF3I	2	121295602	121306440	ENSBTAG00000014388	protein_coding
TMEM234	2	121305656	121311475	ENSBTAG00000009163	protein_coding
DCDC2B	2	121311608	121316391	ENSBTAG00000026696	protein_coding
IQCC	2	121316668	121319689	ENSBTAG00000015103	protein_coding
CCDC28B	2	121319787	121324157	ENSBTAG00000015104	protein_coding
CLDN12	2	121377323	121416305	ENSBTAG00000019486	protein_coding
SHOX	2	121446978	121458374	ENSBTAG00000026788	protein_coding
U6	2	121351995	121352100	ENSBTAG00000046605	snRNA
TMPRSS5	15	24246030	24258672	ENSBTAG00000063291	protein_coding
ZW10	15	24284268	24319385	ENSBTAG00000020066	protein_coding
USP28	15	24336893	24400181	ENSBTAG00000002323	protein_coding
CLDN25	15	24328165	24328857	ENSBTAG00000039149	protein_coding
SLIT3	20	447017	1163732	ENSBTAG00000017746	protein_coding
IQSEC1	22	58592571	58878769	ENSBTAG00000003237	protein_coding
ACAD9	22	58892389	58940024	ENSBTAG00000003242	protein_coding
ALS2CL	22	52793614	52815789	ENSBTAG00000017812	protein_coding
FAM240A	22	52836432	52841404	ENSBTAG00000062614	protein_coding
CRIPTO	22	52867448	52872061	ENSBTAG00000021119	protein_coding
LRRC2	22	52883832	52926453	ENSBTAG00000008013	protein_coding
RTP3	22	52926963	52930809	ENSBTAG00000018767	protein_coding
LTF	22	52934900	52990568	ENSBTAG00000001292	protein_coding
CCR1	22	53199437	53237483	ENSBTAG00000019428	protein_coding
	22	52991916	53222543	ENSBTAG00000031355	protein_coding
CCRL2	22	52998319	53000315	ENSBTAG00000006155	protein_coding
CCR5	22	53024929	53032609	ENSBTAG00000067584	protein_coding
CCR2	22	53041056	53057320	ENSBTAG00000056962	protein_coding
CCR3	22	53134643	53166540	ENSBTAG00000001338	protein_coding
	22	52815938	52820885	ENSBTAG00000071496	lncRNA
	22	52815954	52820898	ENSBTAG00000074759	lncRNA
	22	52931000	52932045	ENSBTAG00000069054	lncRNA
LONRF1	27	24235745	24281816	ENSBTAG00000020084	protein_coding
	27	24222336	24223499	ENSBTAG00000059365	lncRNA
	27	24282931	24286512	ENSBTAG00000066045	lncRNA
	27	24292661	24293782	ENSBTAG00000066043	lncRNA
	27	24330725	24335267	ENSBTAG00000071517	lncRNA
	27	24375165	24383572	ENSBTAG00000069102	lncRNA
	27	24136711	24232205	ENSBTAG00000065743	lncRNA

	27	24295100	24305729	ENSBTAG00000063691	lncRNA
	27	24295972	24384717	ENSBTAG00000065714	lncRNA
CD6	29	37311550	37357284	ENSBTAG00000018367	protein_coding
CD5	29	37422886	37444466	ENSBTAG00000013730	protein_coding
VPS37C	29	37451425	37481097	ENSBTAG00000008064	protein_coding
	29	50930288	51001621	ENSBTAG00000019015	protein_coding
	29	50933710	50983567	ENSBTAG00000048470	protein_coding
	29	51003746	51006379	ENSBTAG00000019017	protein_coding
	29	51049730	51063351	ENSBTAG00000020242	protein_coding

Table S2. Positional candidate genes identified for chamfer defect trait

**Gene**	**Chromossome**	**Genomic Region (Start-End)**		**gene_id**	**gene_biotype**
GRAMD1B	15	33964574	34225319	ENSBTAG00000001410	protein_coding
CLMP	15	33689099	33794265	ENSBTAG00000020046	protein_coding
	15	33942972	33951828	ENSBTAG00000062583	lncRNA
	15	33949760	33955975	ENSBTAG00000058139	lncRNA

Table S3. Positional candidate genes identified for hump defect trait

**Gene**	**Chromosome**	**Genomic Region (Start-End)**		**gene_id**	**gene_biotype**
IQSEC1	22	58592571	58878769	ENSBTAG00000003237	protein_coding
ACAD9	22	58892389	58940024	ENSBTAG00000003242	protein_coding
ATXN1	23	40688296	40830587	ENSBTAG00000019675	protein_coding
GMPR	23	40838557	40899426	ENSBTAG00000015743	protein_coding
	23	40831987	40833465	ENSBTAG00000073862	protein_coding

Table S4. Positional candidate genes identified for the loin defect trait

**Gene**	**Chromosome**	**Genomic Region (Start – End)**		**gene_id**	**gene_biotype**
MARF1	25	14044148	14083629	ENSBTAG00000020387	protein_coding
NDE1	25	14084964	14144211	ENSBTAG00000015986	protein_coding
MYH11	25	14124964	14277425	ENSBTAG00000015988	protein_coding
BMERB1	25	13881271	14026628	ENSBTAG00000011692	protein_coding
**bta-mir-484**	25	14083795	14083857	ENSBTAG00000029976	miRNA
**bta-mir-10161**	25	13990737	13990828	ENSBTAG00000052331	miRNA

Table S5. Significant Gene Ontology (GO) terms (FDR-adjusted p-values < 0.05) identified from candidate genes associated with feet and legs malformation in Nellore cattle.

Functional terms	Source	p_value	Description of function	Genes
**GO:0070098**	GO:BP	3,89E-06	chemokine-mediated signaling pathway	ENSBTAG00000017746,ENSBTAG00000019428,ENSBTAG00000031355,ENSBTAG00000056962,ENSBTAG00000001338
**GO:1990869**	GO:BP	3,89E-06	cellular response to chemokine	ENSBTAG00000017746,ENSBTAG00000019428,ENSBTAG00000031355,ENSBTAG00000056962,ENSBTAG00000001338
**GO:1990868**	GO:BP	3,89E-06	response to chemokine	ENSBTAG00000017746,ENSBTAG00000019428,ENSBTAG00000031355,ENSBTAG00000056962,ENSBTAG00000001338
**GO:0019221**	GO:BP	4,49E-05	cytokine-mediated signaling pathway	ENSBTAG00000017746,ENSBTAG00000019428,ENSBTAG00000031355,ENSBTAG00000056962,ENSBTAG00000001338,ENSBTAG00000019015,ENSBTAG00000048470
**GO:0090026**	GO:BP	1,21E-04	positive regulation of monocyte chemotaxis	ENSBTAG00000019428,ENSBTAG00000031355,ENSBTAG00000056962
**GO:0090025**	GO:BP	1,87E-04	regulation of monocyte chemotaxis	ENSBTAG00000019428,ENSBTAG00000031355,ENSBTAG00000056962
**GO:0002548**	GO:BP	5,39E-04	monocyte chemotaxis	ENSBTAG00000019428,ENSBTAG00000031355,ENSBTAG00000056962
**GO:0071345**	GO:BP	6,47E-04	cellular response to cytokine stimulus	ENSBTAG00000017746,ENSBTAG00000019428,ENSBTAG00000031355,ENSBTAG00000056962,ENSBTAG00000001338,ENSBTAG00000019015,ENSBTAG00000048470
**GO:0045071**	GO:BP	6,63E-04	negative regulation of viral genome replication	ENSBTAG00000001292,ENSBTAG00000019015,ENSBTAG00000048470
**GO:0009605**	GO:BP	6,87E-04	response to external stimulus	ENSBTAG00000017746,ENSBTAG00000001292,ENSBTAG00000019428,ENSBTAG00000031355,ENSBTAG00000067584,ENSBTAG00000056962,ENSBTAG00000001338,ENSBTAG00000018367,ENSBTAG00000019015,ENSBTAG00000048470
**GO:0034097**	GO:BP	7,29E-04	response to cytokine	ENSBTAG00000017746,ENSBTAG00000019428,ENSBTAG00000031355,ENSBTAG00000056962,ENSBTAG00000001338,ENSBTAG00000019015,ENSBTAG00000048470

**GO:1901652**	GO:BP	7,29E-04	response to peptide	ENSBTAG00000017746,ENSBTAG00000019428,ENSBTAG00000031355,ENSBTAG00000056962,ENSBTAG00000001338,ENSBTAG00000019015,ENSBTAG00000048470
**GO:0019722**	GO:BP	1,29E-03	calcium-mediated signaling	ENSBTAG00000019428,ENSBTAG00000031355,ENSBTAG00000067584,ENSBTAG00000001338
**GO:0042330**	GO:BP	1,74E-03	taxis	ENSBTAG00000017746,ENSBTAG00000019428,ENSBTAG00000031355,ENSBTAG00000056962,ENSBTAG00000001338
**GO:0007204**	GO:BP	1,74E-03	positive regulation of cytosolic calcium ion concentration	ENSBTAG00000019428,ENSBTAG00000031355,ENSBTAG00000001338
**GO:0006935**	GO:BP	1,74E-03	chemotaxis	ENSBTAG00000017746,ENSBTAG00000019428,ENSBTAG00000031355,ENSBTAG00000056962,ENSBTAG00000001338
**GO:0006952**	GO:BP	1,82E-03	defense response	ENSBTAG00000001292,ENSBTAG00000019428,ENSBTAG00000031355,ENSBTAG00000056962,ENSBTAG00000001338,ENSBTAG00000018367,ENSBTAG00000019015,ENSBTAG00000048470
**GO:0045069**	GO:BP	1,82E-03	regulation of viral genome replication	ENSBTAG00000001292,ENSBTAG00000019015,ENSBTAG00000048470
**GO:0007165**	GO:BP	1,82E-03	signal transduction	ENSBTAG00000002323,ENSBTAG00000017746,ENSBTAG00000003237,ENSBTAG00000008013,ENSBTAG00000001292,ENSBTAG00000019428,ENSBTAG00000031355,ENSBTAG00000067584,ENSBTAG00000056962,ENSBTAG00000001338,ENSBTAG00000018367,ENSBTAG00000013730,ENSBTAG00000019015,ENSBTAG00000048470
**GO:0048525**	GO:BP	1,82E-03	negative regulation of viral process	ENSBTAG00000001292,ENSBTAG00000019015,ENSBTAG00000048470
**GO:0006955**	GO:BP	1,93E-03	immune response	ENSBTAG00000001292,ENSBTAG00000019428,ENSBTAG00000031355,ENSBTAG00000056962,ENSBTAG00000001338,ENSBTAG00000018367,ENSBTAG00000019015,ENSBTAG00000048470
**GO:0002690**	GO:BP	2,74E-03	positive regulation of leukocyte chemotaxis	ENSBTAG00000019428,ENSBTAG00000031355,ENSBTAG00000056962

**GO:0071677**	GO:BP	2,74E-03	positive regulation of mononuclear cell migration	ENSBTAG00000019428,ENSBTAG00000031355,ENSBTAG00000056962
**GO:0035455**	GO:BP	3,02E-03	response to interferon-alpha	ENSBTAG00000019015,ENSBTAG00000048470
**GO:0023052**	GO:BP	3,97E-03	signaling	ENSBTAG00000002323,ENSBTAG00000017746,ENSBTAG00000003237,ENSBTAG00000008013,ENSBTAG00000001292,ENSBTAG00000019428,ENSBTAG00000031355,ENSBTAG00000067584,ENSBTAG00000056962,ENSBTAG00000001338,ENSBTAG00000018367,ENSBTAG00000013730,ENSBTAG00000019015,ENSBTAG00000048470
**GO:0051851**	GO:BP	3,97E-03	host-mediated perturbation of symbiont process	ENSBTAG00000001292,ENSBTAG00000019015,ENSBTAG00000048470
**GO:0007154**	GO:BP	4,08E-03	cell communication	ENSBTAG00000002323,ENSBTAG00000017746,ENSBTAG00000003237,ENSBTAG00000008013,ENSBTAG00000001292,ENSBTAG00000019428,ENSBTAG00000031355,ENSBTAG00000067584,ENSBTAG00000056962,ENSBTAG00000001338,ENSBTAG00000018367,ENSBTAG00000013730,ENSBTAG00000019015,ENSBTAG00000048470
**GO:0019079**	GO:BP	4,25E-03	viral genome replication	ENSBTAG00000001292,ENSBTAG00000019015,ENSBTAG00000048470
**GO:1903900**	GO:BP	4,26E-03	regulation of viral life cycle	ENSBTAG00000001292,ENSBTAG00000019015,ENSBTAG00000048470
**GO:0035821**	GO:BP	4,31E-03	modulation of process of another organism	ENSBTAG00000001292,ENSBTAG00000019015,ENSBTAG00000048470
**GO:0070887**	GO:BP	4,31E-03	cellular response to chemical stimulus	ENSBTAG00000017746,ENSBTAG00000001292,ENSBTAG00000019428,ENSBTAG00000031355,ENSBTAG00000067584,ENSBTAG00000056962,ENSBTAG00000001338,ENSBTAG00000018367
**GO:0060326**	GO:BP	4,31E-03	cell chemotaxis	ENSBTAG00000019428,ENSBTAG00000031355,ENSBTAG00000056962,ENSBTAG00000001338
**GO:0002376**	GO:BP	4,36E-03	immune system process	ENSBTAG00000001292,ENSBTAG00000019428,ENSBTAG00000031355,ENSBTAG00000056962,ENSBTAG00000001338,ENSBTAG00000018367,

				ENSBTAG00000013730,ENSBTAG00000019015,ENSBTAG00000048470
**GO:0002688**	GO:BP	4,36E-03	regulation of leukocyte chemotaxis	ENSBTAG00000019428,ENSBTAG00000031355,ENSBTAG00000056962
**GO:0046597**	GO:BP	4,51E-03	host-mediated suppression of symbiont invasion	ENSBTAG00000019015,ENSBTAG00000048470
**GO:0006954**	GO:BP	5,50E-03	inflammatory response	ENSBTAG00000019428,ENSBTAG00000031355,ENSBTAG00000056962, ENSBTAG00000001338,ENSBTAG00000018367
**GO:0002684**	GO:BP	5,50E-03	positive regulation of immune system process	ENSBTAG00000001292,ENSBTAG00000019428,ENSBTAG00000031355, ENSBTAG00000056962,ENSBTAG00000018367,ENSBTAG00000013730
**GO:0071675**	GO:BP	5,50E-03	regulation of mononuclear cell migration	ENSBTAG00000019428,ENSBTAG00000031355,ENSBTAG00000056962
**GO:0035456**	GO:BP	5,50E-03	response to interferon-beta	ENSBTAG00000019015,ENSBTAG00000048470
**GO:0002687**	GO:BP	5,50E-03	positive regulation of leukocyte migration	ENSBTAG00000019428,ENSBTAG00000031355,ENSBTAG00000056962
**GO:0050921**	GO:BP	5,70E-03	positive regulation of chemotaxis	ENSBTAG00000019428,ENSBTAG00000031355,ENSBTAG00000056962
**GO:0050792**	GO:BP	5,73E-03	regulation of viral process	ENSBTAG00000001292,ENSBTAG00000019015,ENSBTAG00000048470
**GO:0007166**	GO:BP	5,97E-03	cell surface receptor signaling pathway	ENSBTAG00000017746,ENSBTAG00000001292,ENSBTAG00000019428, ENSBTAG00000031355,ENSBTAG00000056962,ENSBTAG00000001338, ENSBTAG00000018367,ENSBTAG00000019015,ENSBTAG00000048470
**GO:0071222**	GO:BP	5,97E-03	cellular response to lipopolysaccharide	ENSBTAG00000001292,ENSBTAG00000067584,ENSBTAG00000018367
**GO:0007186**	GO:BP	6,77E-03	G protein-coupled receptor signaling pathway	ENSBTAG00000017746,ENSBTAG00000019428,ENSBTAG00000031355, ENSBTAG00000067584,ENSBTAG00000056962,ENSBTAG00000001338
**GO:0071219**	GO:BP	6,94E-03	cellular response to molecule of bacterial origin	ENSBTAG00000001292,ENSBTAG00000067584,ENSBTAG00000018367
**GO:0042221**	GO:BP	7,33E-03	response to chemical	ENSBTAG00000017746,ENSBTAG00000001292,ENSBTAG00000019428, ENSBTAG00000031355,ENSBTAG00000067584,ENSBTAG00000056962, ENSBTAG00000001338,ENSBTAG00000018367,ENSBTAG00000019015, ENSBTAG00000048470

**GO:0051716**	GO:BP	1,06E-02	cellular response to stimulus	ENSBTAG00000002323,ENSBTAG00000017746,ENSBTAG00000003237,ENSBTAG00000008013,ENSBTAG00000001292,ENSBTAG00000019428,ENSBTAG00000031355,ENSBTAG00000067584,ENSBTAG00000056962,ENSBTAG00000001338,ENSBTAG00000018367,ENSBTAG00000013730,ENSBTAG00000019015,ENSBTAG00000048470
**GO:0031663**	GO:BP	1,08E-02	lipopolysaccharide-mediated signaling pathway	ENSBTAG00000001292,ENSBTAG00000018367
**GO:0071216**	GO:BP	1,09E-02	cellular response to biotic stimulus	ENSBTAG00000001292,ENSBTAG00000067584,ENSBTAG00000018367
**GO:0097529**	GO:BP	1,14E-02	myeloid leukocyte migration	ENSBTAG00000019428,ENSBTAG00000031355,ENSBTAG00000056962
**GO:0071674**	GO:BP	1,30E-02	mononuclear cell migration	ENSBTAG00000019428,ENSBTAG00000031355,ENSBTAG00000056962
**GO:0030595**	GO:BP	1,30E-02	leukocyte chemotaxis	ENSBTAG00000019428,ENSBTAG00000031355,ENSBTAG00000056962
**GO:0050870**	GO:BP	1,33E-02	positive regulation of T cell activation	ENSBTAG00000056962,ENSBTAG00000018367,ENSBTAG00000013730
**GO:0050920**	GO:BP	1,36E-02	regulation of chemotaxis	ENSBTAG00000019428,ENSBTAG00000031355,ENSBTAG00000056962
**GO:0002685**	GO:BP	1,39E-02	regulation of leukocyte migration	ENSBTAG00000019428,ENSBTAG00000031355,ENSBTAG00000056962
**GO:0019058**	GO:BP	1,59E-02	viral life cycle	ENSBTAG00000001292,ENSBTAG00000019015,ENSBTAG00000048470
**GO:0032496**	GO:BP	1,59E-02	response to lipopolysaccharide	ENSBTAG00000001292,ENSBTAG00000067584,ENSBTAG00000018367
**GO:1903039**	GO:BP	1,59E-02	positive regulation of leukocyte cell-cell adhesion	ENSBTAG00000056962,ENSBTAG00000018367,ENSBTAG00000013730
**GO:2000464**	GO:BP	1,59E-02	positive regulation of astrocyte chemotaxis	ENSBTAG00000056962
**GO:0043310**	GO:BP	1,59E-02	negative regulation of eosinophil degranulation	ENSBTAG00000056962
**GO:0035705**	GO:BP	1,59E-02	T-helper 17 cell chemotaxis	ENSBTAG00000056962
**GO:2000412**	GO:BP	1,59E-02	positive regulation of thymocyte migration	ENSBTAG00000056962
**GO:0002237**	GO:BP	1,87E-02	response to molecule of bacterial origin	ENSBTAG00000001292,ENSBTAG00000067584,ENSBTAG00000018367
**GO:0090264**	GO:BP	2,09E-02	regulation of immune complex clearance by	ENSBTAG00000056962

			monocytes and macrophages	
**GO:0051251**	GO:BP	2,09E-02	positive regulation of lymphocyte activation	ENSBTAG00000056962,ENSBTAG00000018367,ENSBTAG00000013730
**GO:1900191**	GO:BP	2,09E-02	negative regulation of single-species biofilm formation	ENSBTAG00000001292
**GO:0002436**	GO:BP	2,09E-02	immune complex clearance by monocytes and macrophages	ENSBTAG00000056962
**GO:0098630**	GO:BP	2,09E-02	aggregation of unicellular organisms	ENSBTAG00000001292
**GO:0060337**	GO:BP	2,09E-02	type I interferon-mediated signaling pathway	ENSBTAG00000019015,ENSBTAG00000048470
**GO:0061364**	GO:BP	2,09E-02	apoptotic process involved in luteolysis	ENSBTAG00000017746
**GO:1900190**	GO:BP	2,09E-02	regulation of single-species biofilm formation	ENSBTAG00000001292
**GO:0090265**	GO:BP	2,09E-02	positive regulation of immune complex clearance by monocytes and macrophages	ENSBTAG00000056962
**GO:0071357**	GO:BP	2,09E-02	cellular response to type I interferon	ENSBTAG00000019015,ENSBTAG00000048470
**GO:0044407**	GO:BP	2,09E-02	single-species biofilm formation in or on host organism	ENSBTAG00000001292
**GO:0002434**	GO:BP	2,09E-02	immune complex clearance	ENSBTAG00000056962
**GO:0022409**	GO:BP	2,09E-02	positive regulation of cell-cell adhesion	ENSBTAG00000056962,ENSBTAG00000018367,ENSBTAG00000013730
**GO:0032103**	GO:BP	2,09E-02	positive regulation of response to external stimulus	ENSBTAG00000001292,ENSBTAG00000019428,ENSBTAG00000031355, ENSBTAG00000056962
**GO:2000473**	GO:BP	2,09E-02	positive regulation of hematopoietic stem cell migration	ENSBTAG00000056962
**GO:2000471**	GO:BP	2,09E-02	regulation of hematopoietic stem cell migration	ENSBTAG00000056962
**GO:0033993**	GO:BP	2,09E-02	response to lipid	ENSBTAG00000017746,ENSBTAG00000001292,ENSBTAG00000067584, ENSBTAG00000018367
**GO:0034340**	GO:BP	2,09E-02	response to type I interferon	ENSBTAG00000019015,ENSBTAG00000048470
**GO:2000458**	GO:BP	2,09E-02	regulation of astrocyte chemotaxis	ENSBTAG00000056962

**GO:2000451**	GO:BP	2,09E-02	positive regulation of CD8-positive, alpha-beta T cell extravasation	ENSBTAG00000056962
**GO:0001554**	GO:BP	2,09E-02	luteolysis	ENSBTAG00000017746
**GO:2000449**	GO:BP	2,09E-02	regulation of CD8-positive, alpha-beta T cell extravasation	ENSBTAG00000056962
**GO:0035697**	GO:BP	2,09E-02	CD8-positive, alpha-beta T cell extravasation	ENSBTAG00000056962
**GO:0035700**	GO:BP	2,09E-02	astrocyte chemotaxis	ENSBTAG00000056962
**GO:2000437**	GO:BP	2,09E-02	regulation of monocyte extravasation	ENSBTAG00000056962
**GO:0002682**	GO:BP	2,09E-02	regulation of immune system process	ENSBTAG00000001292,ENSBTAG00000019428,ENSBTAG00000031355,ENSBTAG00000056962,ENSBTAG00000018367,ENSBTAG00000013730
**GO:2000410**	GO:BP	2,09E-02	regulation of thymocyte migration	ENSBTAG00000056962
**GO:1900229**	GO:BP	2,09E-02	negative regulation of single-species biofilm formation in or on host organism	ENSBTAG00000001292
**GO:1900228**	GO:BP	2,09E-02	regulation of single-species biofilm formation in or on host organism	ENSBTAG00000001292
**GO:0044010**	GO:BP	2,09E-02	single-species biofilm formation	ENSBTAG00000001292
**GO:2000439**	GO:BP	2,09E-02	positive regulation of monocyte extravasation	ENSBTAG00000056962
**GO:0042710**	GO:BP	2,09E-02	biofilm formation	ENSBTAG00000001292
**GO:0006950**	GO:BP	2,21E-02	response to stress	ENSBTAG00000002323,ENSBTAG00000001292,ENSBTAG00000019428,ENSBTAG00000031355,ENSBTAG00000056962,ENSBTAG00000001338,ENSBTAG00000018367,ENSBTAG00000019015,ENSBTAG00000048470
**GO:0140888**	GO:BP	2,21E-02	interferon-mediated signaling pathway	ENSBTAG00000019015,ENSBTAG00000048470
**GO:0002696**	GO:BP	2,25E-02	positive regulation of leukocyte activation	ENSBTAG00000056962,ENSBTAG00000018367,ENSBTAG00000013730
**GO:0016032**	GO:BP	2,32E-02	viral process	ENSBTAG00000001292,ENSBTAG00000019015,ENSBTAG00000048470
**GO:0042102**	GO:BP	2,37E-02	positive regulation of T cell proliferation	ENSBTAG00000056962,ENSBTAG00000018367
**GO:0050867**	GO:BP	2,55E-02	positive regulation of cell activation	ENSBTAG00000056962,ENSBTAG00000018367,ENSBTAG00000013730

**GO:0141124**	GO:BP	2,55E-02	intracellular signaling cassette	ENSBTAG00000003237,ENSBTAG00000001292,ENSBTAG00000019428,ENSBTAG00000031355,ENSBTAG00000067584,ENSBTAG00000001338
**GO:1903037**	GO:BP	2,56E-02	regulation of leukocyte cell-cell adhesion	ENSBTAG00000056962,ENSBTAG00000018367,ENSBTAG00000013730
**GO:0050863**	GO:BP	2,73E-02	regulation of T cell activation	ENSBTAG00000056962,ENSBTAG00000018367,ENSBTAG00000013730
**GO:0051414**	GO:BP	2,76E-02	response to cortisol	ENSBTAG00000017746
**GO:0072679**	GO:BP	2,76E-02	thymocyte migration	ENSBTAG00000056962
**GO:0006968**	GO:BP	2,76E-02	cellular defense response	ENSBTAG00000056962
**GO:0043309**	GO:BP	2,76E-02	regulation of eosinophil degranulation	ENSBTAG00000056962
**GO:0050900**	GO:BP	3,00E-02	leukocyte migration	ENSBTAG00000019428,ENSBTAG00000031355,ENSBTAG00000056962
**GO:0040011**	GO:BP	3,25E-02	locomotion	ENSBTAG00000017746,ENSBTAG00000019428,ENSBTAG00000031355,ENSBTAG00000056962,ENSBTAG00000001338
**GO:0007159**	GO:BP	3,33E-02	leukocyte cell-cell adhesion	ENSBTAG00000056962,ENSBTAG00000018367,ENSBTAG00000013730
**GO:0019732**	GO:BP	3,40E-02	antifungal humoral response	ENSBTAG00000001292
**GO:0035696**	GO:BP	3,40E-02	monocyte extravasation	ENSBTAG00000056962
**GO:0070100**	GO:BP	3,40E-02	negative regulation of chemokine-mediated signaling pathway	ENSBTAG00000017746
**GO:0034341**	GO:BP	3,40E-02	response to type II interferon	ENSBTAG00000019015,ENSBTAG00000048470
**GO:0097350**	GO:BP	3,40E-02	neutrophil clearance	ENSBTAG00000056962
**GO:2000409**	GO:BP	3,40E-02	positive regulation of T cell extravasation	ENSBTAG00000056962
**GO:0050896**	GO:BP	3,68E-02	response to stimulus	ENSBTAG00000002323,ENSBTAG00000017746,ENSBTAG00000003237,ENSBTAG00000008013,ENSBTAG00000001292,ENSBTAG00000019428,ENSBTAG00000031355,ENSBTAG00000067584,ENSBTAG00000056962,ENSBTAG00000001338,ENSBTAG00000018367,ENSBTAG00000013730,ENSBTAG00000019015,ENSBTAG00000048470
**GO:0050671**	GO:BP	3,69E-02	positive regulation of lymphocyte proliferation	ENSBTAG00000056962,ENSBTAG00000018367

**GO:0032946**	GO:BP	3,74E-02	positive regulation of mononuclear cell proliferation	ENSBTAG00000056962,ENSBTAG00000018367
**GO:2000407**	GO:BP	3,88E-02	regulation of T cell extravasation	ENSBTAG00000056962
**GO:0043308**	GO:BP	3,88E-02	eosinophil degranulation	ENSBTAG00000056962
**GO:0002887**	GO:BP	3,88E-02	negative regulation of myeloid leukocyte mediated immunity	ENSBTAG00000056962
**GO:0043307**	GO:BP	3,88E-02	eosinophil activation	ENSBTAG00000056962
**GO:0043301**	GO:BP	3,88E-02	negative regulation of leukocyte degranulation	ENSBTAG00000056962
**GO:0035385**	GO:BP	3,88E-02	Roundabout signaling pathway	ENSBTAG00000017746
**GO:1903977**	GO:BP	3,88E-02	positive regulation of glial cell migration	ENSBTAG00000056962
**GO:0002278**	GO:BP	3,88E-02	eosinophil activation involved in immune response	ENSBTAG00000056962
**GO:0097305**	GO:BP	3,89E-02	response to alcohol	ENSBTAG00000017746,ENSBTAG00000067584
**GO:0043207**	GO:BP	4,24E-02	response to external biotic stimulus	ENSBTAG00000001292,ENSBTAG00000067584,ENSBTAG00000018367,ENSBTAG00000019015,ENSBTAG00000048470
**GO:0051707**	GO:BP	4,24E-02	response to other organism	ENSBTAG00000001292,ENSBTAG00000067584,ENSBTAG00000018367,ENSBTAG00000019015,ENSBTAG00000048470
**GO:0070665**	GO:BP	4,30E-02	positive regulation of leukocyte proliferation	ENSBTAG00000056962,ENSBTAG00000018367
**GO:0022407**	GO:BP	4,30E-02	regulation of cell-cell adhesion	ENSBTAG00000056962,ENSBTAG00000018367,ENSBTAG00000013730
**GO:1903238**	GO:BP	4,32E-02	positive regulation of leukocyte tethering or rolling	ENSBTAG00000056962
**GO:0051968**	GO:BP	4,32E-02	positive regulation of synaptic transmission, glutamatergic	ENSBTAG00000056962
**GO:0043615**	GO:BP	4,32E-02	astrocyte cell migration	ENSBTAG00000056962
**GO:0045785**	GO:BP	4,32E-02	positive regulation of cell adhesion	ENSBTAG00000056962,ENSBTAG00000018367,ENSBTAG00000013730
**GO:0002447**	GO:BP	4,32E-02	eosinophil mediated immunity	ENSBTAG00000056962

**GO:0008284**	GO:BP	4,34E-02	positive regulation of cell population proliferation	ENSBTAG00000001292,ENSBTAG00000056962,ENSBTAG00000001338,ENSBTAG00000018367
**GO:0071396**	GO:BP	4,43E-02	cellular response to lipid	ENSBTAG00000001292,ENSBTAG00000067584,ENSBTAG00000018367
**GO:0051249**	GO:BP	4,43E-02	regulation of lymphocyte activation	ENSBTAG00000056962,ENSBTAG00000018367,ENSBTAG00000013730
**GO:0009607**	GO:BP	4,43E-02	response to biotic stimulus	ENSBTAG00000001292,ENSBTAG00000067584,ENSBTAG00000018367,ENSBTAG00000019015,ENSBTAG00000048470
**GO:0032680**	GO:BP	4,43E-02	regulation of tumor necrosis factor production	ENSBTAG00000001292,ENSBTAG00000056962
**GO:0032640**	GO:BP	4,43E-02	tumor necrosis factor production	ENSBTAG00000001292,ENSBTAG00000056962
**GO:1903555**	GO:BP	4,53E-02	regulation of tumor necrosis factor superfamily cytokine production	ENSBTAG00000001292,ENSBTAG00000056962
**GO:0071706**	GO:BP	4,53E-02	tumor necrosis factor superfamily cytokine production	ENSBTAG00000001292,ENSBTAG00000056962
**GO:0035556**	GO:BP	4,55E-02	intracellular signal transduction	ENSBTAG00000002323,ENSBTAG00000003237,ENSBTAG00000001292,ENSBTAG00000019428,ENSBTAG00000031355,ENSBTAG00000067584,ENSBTAG00000001338
**GO:0035701**	GO:BP	4,64E-02	hematopoietic stem cell migration	ENSBTAG00000056962
**GO:0031665**	GO:BP	4,64E-02	negative regulation of lipopolysaccharide-mediated signaling pathway	ENSBTAG00000001292
**GO:0070099**	GO:BP	4,64E-02	regulation of chemokine-mediated signaling pathway	ENSBTAG00000017746
**GO:0042129**	GO:BP	4,96E-02	regulation of T cell proliferation	ENSBTAG00000056962,ENSBTAG00000018367
**GO:0009897**	GO:CC	2,67E-05	external side of plasma membrane	ENSBTAG00000019428,ENSBTAG00000031355,ENSBTAG00000067584,ENSBTAG00000056962,ENSBTAG00000001338,ENSBTAG00000013730
**GO:0098552**	GO:CC	1,42E-04	side of membrane	ENSBTAG00000019428,ENSBTAG00000031355,ENSBTAG00000067584,ENSBTAG00000056962,ENSBTAG00000001338,ENSBTAG00000013730

**GO:0009986**	GO:CC	1,42E-04	cell surface	ENSBTAG00000001292,ENSBTAG00000019428,ENSBTAG00000031355,ENSBTAG00000067584,ENSBTAG00000056962,ENSBTAG00000001338,ENSBTAG00000013730
**GO:0016493**	GO:MF	5,10E-10	C-C chemokine receptor activity	ENSBTAG00000019428,ENSBTAG00000031355,ENSBTAG00000067584,ENSBTAG00000056962,ENSBTAG00000001338
**GO:0019957**	GO:MF	5,10E-10	C-C chemokine binding	ENSBTAG00000019428,ENSBTAG00000031355,ENSBTAG00000067584,ENSBTAG00000056962,ENSBTAG00000001338
**GO:0001637**	GO:MF	1,95E-09	G protein-coupled chemoattractant receptor activity	ENSBTAG00000019428,ENSBTAG00000031355,ENSBTAG00000067584,ENSBTAG00000056962,ENSBTAG00000001338
**GO:0004950**	GO:MF	1,95E-09	chemokine receptor activity	ENSBTAG00000019428,ENSBTAG00000031355,ENSBTAG00000067584,ENSBTAG00000056962,ENSBTAG00000001338
**GO:0019956**	GO:MF	3,05E-09	chemokine binding	ENSBTAG00000019428,ENSBTAG00000031355,ENSBTAG00000067584,ENSBTAG00000056962,ENSBTAG00000001338
**GO:0071791**	GO:MF	3,15E-08	chemokine (C-C motif) ligand 5 binding	ENSBTAG00000019428,ENSBTAG00000031355,ENSBTAG00000067584
**GO:0035717**	GO:MF	3,15E-08	chemokine (C-C motif) ligand 7 binding	ENSBTAG00000019428,ENSBTAG00000031355,ENSBTAG00000056962
**GO:0004896**	GO:MF	4,49E-07	cytokine receptor activity	ENSBTAG00000019428,ENSBTAG00000031355,ENSBTAG00000067584,ENSBTAG00000056962,ENSBTAG00000001338
**GO:0140375**	GO:MF	1,20E-06	immune receptor activity	ENSBTAG00000019428,ENSBTAG00000031355,ENSBTAG00000067584,ENSBTAG00000056962,ENSBTAG00000001338
**GO:0019955**	GO:MF	1,57E-06	cytokine binding	ENSBTAG00000019428,ENSBTAG00000031355,ENSBTAG00000067584,ENSBTAG00000056962,ENSBTAG00000001338
**GO:0004930**	GO:MF	3,93E-03	G protein-coupled receptor activity	ENSBTAG00000019428,ENSBTAG00000031355,ENSBTAG00000067584,ENSBTAG00000056962,ENSBTAG00000001338
**GO:0001530**	GO:MF	4,33E-03	lipopolysaccharide binding	ENSBTAG00000001292,ENSBTAG00000018367

**GO:0048020**	GO:MF	5,46E-03	CCR chemokine receptor binding	ENSBTAG00000006155,ENSBTAG00000056962
**GO:0035716**	GO:MF	1,04E-02	chemokine (C-C motif) ligand 12 binding	ENSBTAG00000056962
**GO:0035715**	GO:MF	1,04E-02	chemokine (C-C motif) ligand 2 binding	ENSBTAG00000056962
**GO:0042379**	GO:MF	1,25E-02	chemokine receptor binding	ENSBTAG00000006155,ENSBTAG00000056962
**GO:0031727**	GO:MF	1,74E-02	CCR2 chemokine receptor binding	ENSBTAG00000056962
**GO:0140912**	GO:MF	1,74E-02	membrane destabilizing activity	ENSBTAG00000001292
**GO:0004888**	GO:MF	1,86E-02	transmembrane signaling receptor activity	ENSBTAG00000019428,ENSBTAG00000031355,ENSBTAG00000067584,ENSBTAG00000056962,ENSBTAG00000001338
**GO:0070891**	GO:MF	2,23E-02	lipoteichoic acid binding	ENSBTAG00000018367
**GO:0070991**	GO:MF	2,23E-02	medium-chain fatty acyl-CoA dehydrogenase activity	ENSBTAG00000003242
**GO:0004466**	GO:MF	2,84E-02	long-chain fatty acyl-CoA dehydrogenase activity	ENSBTAG00000003242
**GO:0048495**	GO:MF	4,06E-02	Roundabout binding	ENSBTAG00000017746
**GO:0052890**	GO:MF	4,98E-02	oxidoreductase activity, acting on the CH-CH group of donors, with a flavin as acceptor	ENSBTAG00000003242
**GO:0003995**	GO:MF	4,98E-02	acyl-CoA dehydrogenase activity	ENSBTAG00000003242
**KEGG:04061**	KEGG	6,28E-06	Viral protein interaction with cytokine and cytokine receptor	ENSBTAG00000019428,ENSBTAG00000031355,ENSBTAG00000067584,ENSBTAG00000056962,ENSBTAG00000001338
**KEGG:04060**	KEGG	5,62E-05	Cytokine-cytokine receptor interaction	ENSBTAG00000019428,ENSBTAG00000031355,ENSBTAG00000067584,ENSBTAG00000056962,ENSBTAG00000001338,ENSBTAG00000020242
**KEGG:04062**	KEGG	8,13E-05	Chemokine signaling pathway	ENSBTAG00000019428,ENSBTAG00000031355,ENSBTAG00000067584,ENSBTAG00000056962,ENSBTAG00000001338
**KEGG:05167**	KEGG	1,64E-03	Kaposi sarcoma-associated herpesvirus infection	ENSBTAG00000019428,ENSBTAG00000031355,ENSBTAG00000067584,ENSBTAG00000001338
**KEGG:05163**	KEGG	2,28E-03	Human cytomegalovirus infection	ENSBTAG00000019428,ENSBTAG00000031355,ENSBTAG00000067584,ENSBTAG00000001338

**KEGG:04514**	KEGG	8,39E-03	Cell adhesion molecules	ENSBTAG00000019486,ENSBTAG00000039149,ENSBTAG00000018367
**KEGG:03260**	KEGG	2,40E-02	Virion - Human immunodeficiency virus	ENSBTAG00000067584
**KEGG:04144**	KEGG	2,40E-02	Endocytosis	ENSBTAG00000003237,ENSBTAG00000067584,ENSBTAG00000008064
**KEGG:04670**	KEGG	4,69E-02	Leukocyte transendothelial migration	ENSBTAG00000019486,ENSBTAG00000039149

Abbreviations: GO:BP, Gene Ontology Biological Process; GO:MF, Gene Ontology Molecular Function; KEGG, Kyoto Encyclopedia of Genes and Genomes pathway.

Table S6. Significant Gene Ontology (GO) terms (FDR-adjusted p-values < 0.05) identified from candidate genes associated with chamfer defect in Nellore cattle.

**Functional terms**	**Source**	**p_value**	**Description of function**	**Genes**
**GO:0099170**	GO:BP	0,024	postsynaptic modulation of chemical synaptic transmission	ENSBTAG00000020046
**GO:0036315**	GO:BP	0,024	cellular response to sterol	ENSBTAG00000001410
**GO:0070723**	GO:BP	0,024	response to cholesterol	ENSBTAG00000001410
**GO:0036314**	GO:BP	0,024	response to sterol	ENSBTAG00000001410
**GO:0071397**	GO:BP	0,024	cellular response to cholesterol	ENSBTAG00000001410
**GO:0097306**	GO:BP	0,046	cellular response to alcohol	ENSBTAG00000001410
**GO:0055092**	GO:BP	0,046	sterol homeostasis	ENSBTAG00000001410
**GO:0042632**	GO:BP	0,046	cholesterol homeostasis	ENSBTAG00000001410
**GO:0140268**	GO:CC	0,045	endoplasmic reticulum-plasma membrane contact site	ENSBTAG00000001410
**GO:0001786**	GO:MF	0,009	phosphatidylserine binding	ENSBTAG00000001410
**GO:0120020**	GO:MF	0,009	cholesterol transfer activity	ENSBTAG00000001410
**GO:0120015**	GO:MF	0,009	sterol transfer activity	ENSBTAG00000001410
**GO:0120013**	GO:MF	0,009	lipid transfer activity	ENSBTAG00000001410
**GO:0070300**	GO:MF	0,009	phosphatidic acid binding	ENSBTAG00000001410
**GO:0043178**	GO:MF	0,009	alcohol binding	ENSBTAG00000001410
**GO:0072341**	GO:MF	0,009	modified amino acid binding	ENSBTAG00000001410
**GO:0015485**	GO:MF	0,009	cholesterol binding	ENSBTAG00000001410
**GO:0032934**	GO:MF	0,009	sterol binding	ENSBTAG00000001410
**GO:0005496**	GO:MF	0,010	steroid binding	ENSBTAG00000001410
**GO:0005319**	GO:MF	0,015	lipid transporter activity	ENSBTAG00000001410
**GO:0005543**	GO:MF	0,036	phospholipid binding	ENSBTAG00000001410

Abbreviations: GO:BP, Gene Ontology Biological Process; GO:MF, Gene Ontology Molecular Function; KEGG, Kyoto Encyclopedia of Genes and Genomes pathway.

Table S7. Significant Gene Ontology (GO) terms (FDR-adjusted p-values < 0.05) identified from candidate genes associated with hump defect in Nellore cattle.

Functional terms	Source	p_value	Description of function	Genes
**GO:0032011**	GO:BP	0,049	ARF protein signal transduction	ENSBTAG00000003237
**GO:0032012**	GO:BP	0,049	regulation of ARF protein signal transduction	ENSBTAG00000003237
**GO:0051791**	GO:BP	0,049	medium-chain fatty acid metabolic process	ENSBTAG00000003242
**GO:0042405**	GO:CC	0,048	nuclear inclusion body	ENSBTAG00000019675
**GO:0003920**	GO:MF	0,014	GMP reductase activity	ENSBTAG00000015743
**GO:0070991**	GO:MF	0,014	medium-chain fatty acyl-CoA dehydrogenase activity	ENSBTAG00000003242
**GO:0004466**	GO:MF	0,014	long-chain fatty acyl-CoA dehydrogenase activity	ENSBTAG00000003242
**GO:0046857**	GO:MF	0,014	oxidoreductase activity, acting on other nitrogenous compounds as donors, with NAD or NADP as acceptor	ENSBTAG00000015743
**GO:0034046**	GO:MF	0,016	poly(G) binding	ENSBTAG00000019675
**GO:0052890**	GO:MF	0,016	oxidoreductase activity, acting on the CH-CH group of donors, with a flavin as acceptor	ENSBTAG00000003242
**GO:0003995**	GO:MF	0,016	acyl-CoA dehydrogenase activity	ENSBTAG00000003242
**GO:0016661**	GO:MF	0,022	oxidoreductase activity, acting on other nitrogenous compounds as donors	ENSBTAG00000015743
**GO:0008266**	GO:MF	0,024	poly(U) RNA binding	ENSBTAG00000019675
**GO:0008187**	GO:MF	0,024	poly-pyrimidine tract binding	ENSBTAG00000019675
**GO:0070717**	GO:MF	0,025	poly-purine tract binding	ENSBTAG00000019675
**GO:0016491**	GO:MF	0,035	oxidoreductase activity	ENSBTAG00000003242, ENSBTAG00000015743
**GO:0016627**	GO:MF	0,045	oxidoreductase activity, acting on the CH-CH group of donors	ENSBTAG00000003242

Abbreviations: GO:BP, Gene Ontology Biological Process; GO:MF, Gene Ontology Molecular Function; KEGG, Kyoto Encyclopedia of Genes and Genomes pathway.

Table S8. Significant Gene Ontology (GO) terms (FDR-adjusted p-values < 0.05) identified from candidate genes associated with loin defect in Nellore cattle

Functional terms	Source	p_value	Description of function	Genes
**GO:0021822**	GO:BP	0,018	negative regulation of cell motility involved in cerebral cortex radial glia guided migration	ENSBTAG00000011692
**GO:0021987**	GO:BP	0,018	cerebral cortex development	ENSBTAG00000015986,ENSBTAG00000011692

**GO:0031109**	GO:BP	0,018	microtubule polymerization or depolymerization	ENSBTAG00000015986,ENSBTAG00000011692
**GO:0097435**	GO:BP	0,018	supramolecular fiber organization	ENSBTAG00000015986,ENSBTAG00000015988,ENSBTAG00000011692
**GO:0021543**	GO:BP	0,024	pallium development	ENSBTAG00000015986,ENSBTAG00000011692
**GO:0021537**	GO:BP	0,043	telencephalon development	ENSBTAG00000015986,ENSBTAG00000011692
**GO:1903976**	GO:BP	0,043	negative regulation of glial cell migration	ENSBTAG00000011692
**GO:0021814**	GO:BP	0,044	cell motility involved in cerebral cortex radial glia guided migration	ENSBTAG00000011692
**GO:0005871**	GO:CC	0,040	kinesin complex	ENSBTAG00000015986
**GO:0016460**	GO:CC	0,040	myosin II complex	ENSBTAG00000015988
**GO:0031616**	GO:CC	0,040	spindle pole centrosome	ENSBTAG00000015986
**GO:0032982**	GO:CC	0,040	myosin filament	ENSBTAG00000015988
**GO:0000146**	GO:MF	0,030	microfilament motor activity	ENSBTAG00000015988
**GO:0008092**	GO:MF	0,030	cytoskeletal protein binding	ENSBTAG00000015986,ENSBTAG00000015988
**GO:0008307**	GO:MF	0,030	structural constituent of muscle	ENSBTAG00000015988
**KEGG:04810**	KEGG	0,033	Regulation of actin cytoskeleton	ENSBTAG00000015988
**KEGG:04814**	KEGG	0,033	Motor proteins	ENSBTAG00000015988
**KEGG:04530**	KEGG	0,033	Tight junction	ENSBTAG00000015988
**KEGG:04270**	KEGG	0,033	Vascular smooth muscle contraction	ENSBTAG00000015988

Abbreviations: GO:BP, Gene Ontology Biological Process; GO:MF, Gene Ontology Molecular Function; KEGG, Kyoto Encyclopedia of Genes and Genomes pathway.
